# 10-Gingerol Inhibits Ovarian Cancer Cell Growth by Inducing G2 Arrest

**DOI:** 10.15171/apb.2019.080

**Published:** 2019-10-24

**Authors:** Andrea Rasmussen, Kaylee Murphy, David W. Hoskin

**Affiliations:** ^1^Department of Pathology, Faculty of Medicine, Dalhousie University, Halifax, Nova Scotia B3H 4R2, Canada.; ^2^Department of Microbiology and Immunology, Faculty of Medicine, Dalhousie University, Halifax, Nova Scotia B3H 4R2, Canada.; ^3^Department of Surgery, Faculty of Medicine, Dalhousie University, Halifax, Nova Scotia B3H 4R2, Canada.

**Keywords:** Cell cycle, Proliferation, Ginger, 10-Gingerol, Ovarian cancer

## Abstract

***Purpose:*** Gingerol homologs found in the rhizomes of ginger plants have the potential to benefit human health, including the prevention and treatment of cancer. This study evaluated the effect of 10-gingerol on ovarian cancer cell (HEY, OVCAR3, and SKOV-3) growth.

***Methods:*** Cell growth was measured by MTT assays, flow cytometry was used to assess cell proliferation, cytotoxicity and cell cycle progression, and western blotting was used to measure cyclin protein expression.

*** Results:*** Ovarian cancer cells that were treated with 10-gingerol experienced a time- and dose-dependent decrease in cell number, which was due to a reduction in cell proliferation rather than a cytotoxic effect. Reduced proliferation of 10-gingerol-treated ovarian cancer cells was associated with an increased percentage of cells in G2 phase of the cell cycle and a corresponding reduction in the percentage of cells in G1. Ovarian cancer cells also showed decreased cyclin A, B1, and D3 expression following exposure to 10-gingerol.

***Conclusion:*** These findings revealed that 10-gingerol caused a G2 arrest-associated suppression of ovarian cancer cell growth, which may be exploited in the management of ovarian cancer.

## Introduction

**Figure 1:**
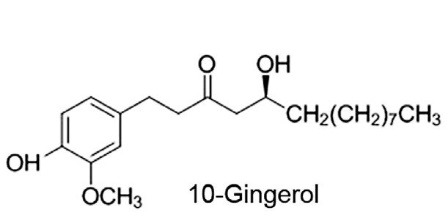
Chemical structure of 10-gingerol.

Ginger root has a tradition of use as a remedy for numerous ailments that include nausea, loss of appetite, cramps, diarrhea, heartburn, migraines, colds, influenza, and arthritis.^1^ Ginger also contains a number of bioactive compounds with anticancer activities.^2^ Gingerols, which are a series of pungent phenolic homologs that differ in unbranched alkyl chain length, are important biologically active constituents of the rhizomes of ginger.^3^ Numerous laboratory studies attest to the anticancer activities of 6-gingerol, which has been the focus of most research.^4^ For example, 6-gingerol induces G1 phase cell cycle arrest and death of colorectal cancer cells, inhibits hepatocarcinoma cell motility and invasion, and the activity of metastasis-promoting matrix metalloproteinase-9.^5,6^ Anticancer activities have also been attributed to 10-gingerol (Figure 1), which we have recently shown to be is a potent inhibitor of breast cancer cell growth, acting via blockade of cellular proliferation and induction of programmed cell death.^7^ In a mouse model of triple-negative breast cancer, orthotopic tumor growth and metastasis to multiple organs is also inhibited by 10-gingerol.^8^ However, information regarding the biological activity of 10-gingerol in ovarian cancer cells is limited. Ovarian cancer is the leading cause of death in gynecologic cancer patients, and is in urgent need of new treatments because of its aggressive nature, high rate of recurrence, and proclivity to develop resistance to chemotherapeutic drugs.^9,10^ In this study, we assessed the impact of 10-gingerol on the growth of HEY, OVCAR3, and SKOV-3 ovarian cancer cell lines, as well as the mechanism of action of 10-gingerol in HEY cells.

## Materials and Methods

### Materials

10-Gingerol (purity >98%) was from Chengdu Biopurify Phytochemicals Ltd. (Chengdu, Sichuan, China). RNase A was from Qiagen (Hilden, Germany. Annexin V-FLUOS was from Roche Applied Sciences (Laval, QC). Oregon Green 488 dye was from Invitrogen (Burlington, ON). Triton X-100, aprotinin, dimethyl sulfoxide (DMSO), methylthiazolyldiphenyl-tetrazolium bromide (MTT), leupeptin, NP-40, pepstatin, phenylarsine oxide, phenylmethyl sulphonyl fluoride, phosphate buffered saline (PBS), propidium iodide (PI), sodium deoxycholate, and sodium fluoride were from Sigma-Aldrich Canada (Oakville, ON). Acrylamide, ammonium persulfate, dithiothreitol, ethylene glycol tetraacetic acid, glycine, paraformaldehyde, sodium dodecyl sulfate (SDS), tetramethylethylenediamine, NaCl, Tris-HCl, and Tween-20 were from Bio-Shop Canada (Burlington, ON). Ethylenediaminetetraacetic acid was from EM Industries (Hawthorne, NY). Sodium orthovanadate was from EMD Chemicals (Gibbstown, NJ). Bromophenol blue was from New England Biolabs (Ipswich, MA). Anti-human cyclin B1 rabbit antibody and anti-human cyclin D3 mouse monoclonal antibody, as well as horseradish peroxidase (HRP)-conjugated goat antibodies against mouse IgG were from Cell Signaling Technology (Beverly, MA). Mouse monoclonal antibody against human cyclin A was from EMD Millipore (Billerica, MA). Anti-human β-actin rabbit antibody and HRP-conjugated goat antibodies against rabbit IgG were from Santa Cruz Biotechnology (Dallas, TX).

### Cell culture

HEY cells were provided by Dr. Mark Nachtigal (University of Manitoba, Winnipeg, MB). Dr. Graham Dellaire (Dalhousie University, Halifax, NS) provided OVCAR3 cells. SKOV-3 cells were obtained from Immunovaccine (Halifax, NS). Short tandem repeat profiling conducted by ATCC (Manassas, VA) was used to authenticate cell lines. Cells were cultured at 37°C in a 10% CO_2_ humidified atmosphere in Dulbecco Modified Eagle Medium (DMEM; Sigma-Aldrich Canada containing 10% heat-inactivated (at 56°C for 30 min) fetal bovine serum, 2mM L-glutamine, 5mM HEPES buffer (pH 7.4), 100 U/mL penicillin and 100 mg/mL streptomycin (all from Invitrogen). Fully supplemented DMEM is referred to as complete DMEM. TrypLE (Invitrogen) was used to passage all cell cultures.

### Cell growth inhibition assessment

HEY, OVCAR3, or SKOV-3 cells were added to a 96-well flat-bottom microtiter plate (5 × 10^3^ cells/well), allowed to form monolayers, and then treated with medium alone, vehicle (DMSO), or different concentrations of 10-gingerol for 24, 48, or 72 h. MTT was added (final concentration, 0.5 μg/mL) for the last 2 h of culture, after which culture supernatant was removed and 0.1 ml DMSO was added to each well to dissolve formazan crystals. Absorbance at 570 nm was measured with an ASYS Expert 96 plate reader with DigiRead software (Biochrom, Cambridge, UK). In cell-free medium, MTT was not reduced by 10-gingerol.

### Cell proliferation assay

Oregon Green 488 dye at 1.25 µM was added to HEY cells (1.5 × 10^4^ cells/well) in a 6-well plate. After incubation for 45 min at 37°C, cells were washed and treated with medium alone, vehicle (DMSO), or 10-gingerol for 72 h. Before culture, a sample of cells was fixed with 1% [w/v] paraformaldehyde to establish baseline fluorescence. Fixed cells were stored at 4°C. At the end of culture cells were fixed and a minimum of 1 × 10^4^ counts per sample were analyzed with a FACSCaliber flow cytometer and Cell Quest^TM^ software (version 3.3; BD Biosciences, Mississauga, ON). The formula, MCF_baselinel_ = (2^n^)(MCF_sample_), where MCF_baseline_ is the baseline mean channel fluorescence (MCF) was used to calculate the number of cell divisions (n).

### Cytotoxicity assay

Approximately 3 × 10^5^ HEY cells were placed into 25 cm^2^ flasks and treated with medium alone, vehicle (DMSO), or 10-gingerol for 24 h. Cell were then stained with Annexin V-FLUOS, as recommended by the supplier, at 1 µg/mL in flow cytometry buffer (10 mM HEPES, 140 mM NaCl, 5 mM CaCl_2_). Then, cells were washed and resuspended in flow cytometry buffer. The percentage of healthy and dead (apoptotic or necrotic) cells was determined by flow cytometry.

### Cell cycle analysis

HEY cells were synchronized in G0 by serum-starvation for 24 h. Cells were then placed in complete DMEM and added to 6-well plates at 2.5 × 10^5^ cells/well. After 72 h treatment with vehicle (DMSO) or 10-gingerol, cells were placed in ice-cold PBS, followed by the drop-wise addition of ice-cold 70% ethanol. After storage at -20°C for at least 24 h, cells were stained with PI at 0.02 mg/ml in PBS containing 0.1% [v/v] Triton X-100 and 0.2 mg/ml DNase-free RNase A. Flow cytometry and ModFit LT 3.0 software (Verity Software House, Topsham, ME) was used to determine the percentage of cells in different phases of the cell cycle.

### Western blot analysis

HEY cells were placed into 6-well plates at 2.5 × 10^5^ cells/well and treated with vehicle (DMSO) or 10-gingerol for 48 h. Cells were then collected and placed in ice-cold RIPA buffer (1% NP-40, 0.5% sodium deoxycholate, 0.1% SDS, 20 mM Tris-HCl, 150 mM NaCl, 1 mM ethylenediaminetetraacetic acid, and 1 mM ethylene glycol tetraacetic acid) containing a protease inhibitor cocktail (5 µg/mL leupeptin, 5 µg/mL pepstatin, 10 µg/mL aprotinin, 100 µM sodium orthovanadate, 10 mM sodium fluoride, 10 µM phenylarsine oxide, 1 mM dithiothreitol, 5 µM phenylmethyl sulphonyl fluoride) for 25 min. The resulting lysates were clarified by centrifugation and stored at -80°C.Protein was quantified by Bradford assay and 10 µg protein in sample buffer (200 nM Tris-HCl [pH 6.8], 30% glycerol [v/v], 6% SDS [w/v], 15% β-mercaptoethanol [v/v], and 0.01% bromophenol blue [w/v]) were loaded onto 10% Tris-HCl polyacrylamide gels containing 375 mM Tris-HCl [pH 8.8], 0.1% SDS [w/v], 0.1% ammonium persulfate [w/v], and 0.15% tetramethylethylenediamine [v/v]. Gels were run for 1 h at 200 V in running buffer (20 mM Tris-HCl [pH 8.3], 200mM glycine, and 0.1% SDS [v/v]). An iBlot (Invitrogen) was used to transfer protein onto a nitrocellulose membrane, which was then blocked for 1 h in 5% skim milk powder [w/v] dissolved in TTBS (20 mM Tris-HCl [pH 7.6], 200 mM NaCl, and 0.05% Tween 20 [v/v]). Membranes were incubated overnight at 4°C with primary antibodies, at the manufacturer’s recommended concentrations, in TTBS containing 5% skim milk powder, then washed and incubated at room temperature for 1 h with the appropriate HRP-conjugated secondary antibody (1:10000) in TTBS containing 5% skim milk powder. Blots were washed and protein bands were visualized with enhanced chemiluminescence reagent (GE Healthcare Canada, Mississauga ON) using the Chemidoc Touch (BioRad, Mississauga, ON).

### Statistics

Microsoft Excel and GraphPad Prism (version 7) were used for data analysis. Analysis of variance (ANOVA) and the Tukey-Kramer comparisons test was used to assess statistical significance (*P* < 0.05).

## Results and Discussion

### Inhibitory effect of 10-gingerol on the growth of ovarian cancer cells

**Figure 2:**
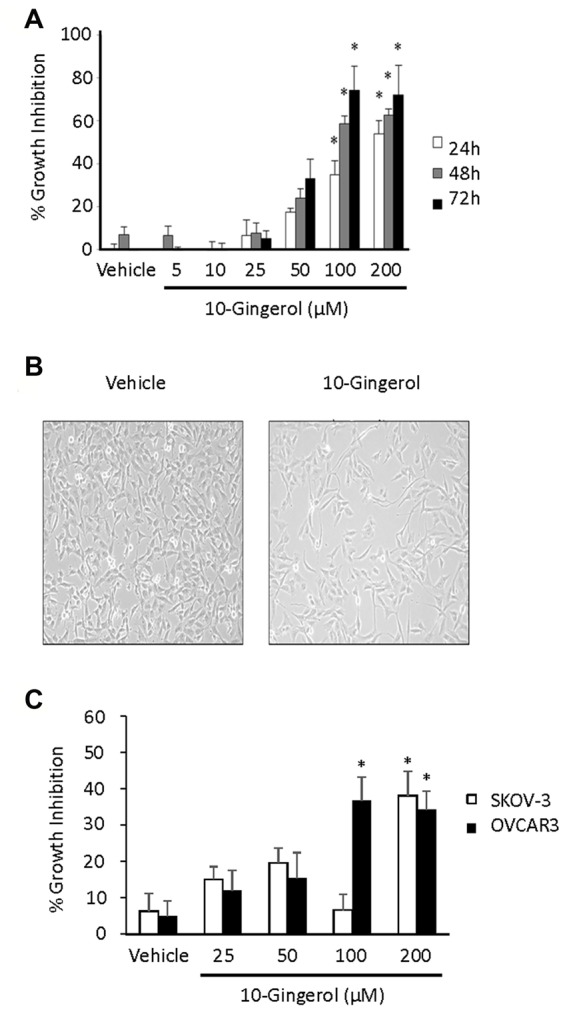
Inhibition of ovarian cancer cell growth by 10-gingerol.(**A**) HEY cells were cultured for 24, 48, or 72 h in the presence of vehicle (DMSO) or the indicated concentrations of 10-gingerol. Relative cell number at the end of culture was determined by MTT assay. Data are shown as the mean percent growth inhibition ± the standard error of the mean (SEM) of 3 independent experiments; (**B**) HEY cells were photographed after 24 h treatment with vehicle or 100 µM 10-gingerol, × 100. (**C**)OVCAR3 and SKOV-3 cells were cultured for 72 h in the presence of vehicle or the indicated concentrations of 10-gingerol, and mean percent growth inhibition ± SEM in 5 independent experiments was determined as with HEY cells. Asterisk denotes *P* < 0.05 compared to the vehicle control.

The impact of 10-gingerol on the growth of 3 different ovarian cancer cell lines was assessed using MTT assays. We observed a time- and dose-dependent inhibition of the growth of HEY ovarian cancer cells; 34 ± 6% growth inhibition (*P* < 0.05 vs. vehicle control) at 24 h by 100 µM 10-gingerol, 71 ± 14% growth inhibition (*P* < 0.05 vs. vehicle control) at 72 h by 200 µM 10-gingerol (Figure 2A). Visual examination of HEY cell cultures showed an approximate 50% reduction in cell number, relative to vehicle-treated cultures, after 24 h exposure to 100 µM 10-gingerol (Figure 2B). This was consistent with the results from MTT assays. A growth-inhibitory effect of 10-gingerol was also observed in OVCAR3 (33 ± 5% growth inhibition, *P* < 0.05 vs. vehicle control) and SKOV-3 (38 ± 7% growth inhibition, *P* < 0.05 vs. vehicle control) ovarian cancer cell cultures after 72 h exposure to 200 µM 10-gingerol (Figure 2C). Subsequent investigations used HEY cells because this ovarian cancer cell line was most sensitive to growth inhibition by 10-gingerol. Decreased ovarian cancer cell growth in the presence of 10-gingerol was consistent with an earlier report that ginger extract, which contains gingerols plus other bioactive compounds, suppresses the growth of A2780, SKOV-3 and CaOV3 ovarian cancer cell lines, as assessed by sulforhodamine B assays.^11^ Importantly, the same study implies a selective effect on malignant cells since ginger extracts do not impact the growth of normal human surface ovarian epithelial cells.

### Cytostatic effect of 10-gingerol on ovarian cancer cells

**Figure 3:**
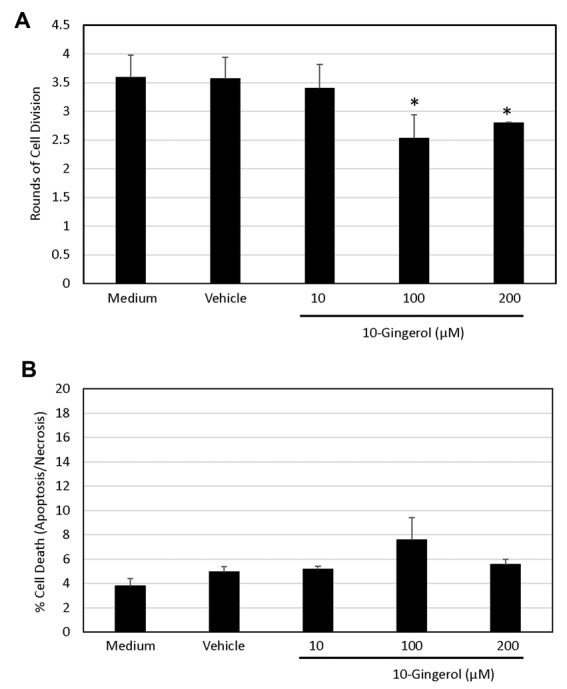
Anti-proliferative effect of 10-gingerol on ovarian cancer cells. **(A)** Oregon Green 488-stained HEY cells were cultured for 72 h in the absence or presence of the indicated concentrations of 10-gingerol, and flow cytometric analysis was used to determine the mean number of divisions ± SEM in 5 independent experiments; asterisk denotes p < 0.05 compared to the vehicle control. **(B)** HEY cells were cultured for 24 h in the absence or presence of the indicated concentrations of [10]-gingerol, then stained with Annexin V-FLUOS and PI for determination of the proportion of viable cells and cells in early apoptosis or late apoptosis/necrosis by flow cytometry. Data are shown as the mean percent cell death ± SEM.

Since MTT assays do not differentiate between cytostatic and cytotoxic effects, we stained HEY cells with Oregon Green 488 dye or Annexin V-FLUOS and PI in order to determine the effect of 10-gingerol on cell proliferation and cell viability, respectively, by flow cytometry. Figure 3A shows that exposure of HEY cells to 100 or 200 µM 10-gingerol for 72 h resulted in fewer rounds of cell division (30% and 28% reduction in rounds of cell division, respectively; *P* < 0.05 vs. vehicle control). A similar inhibitory effect on the proliferation of triple-negative breast cancer cells was seen when these cells were treated with 10-gingerol.^7^ In contrast, Figure 3B shows that there was no loss of viability when HEY cells cultured for 24 h in the presence of 200 µM 10-gingerol (4 ± 1% apoptotic plus necrotic cells in vehicle-treated culture vs. 5 ± 1% apoptotic plus necrotic cells in 10-gingerol-treated cultures, *P* > 0.05). This finding was in sharp contrast to the apoptotic effect of 10-gingerol on triple-negative mammary carcinoma cells after only 24 h treatment.^7,8^ The effect of 10-gingerol therefore appears to differ between ovarian and breast cancer cells.

### G2 arrest in ovarian cancer cells mediated by 10-gingerol

**Figure 4:**
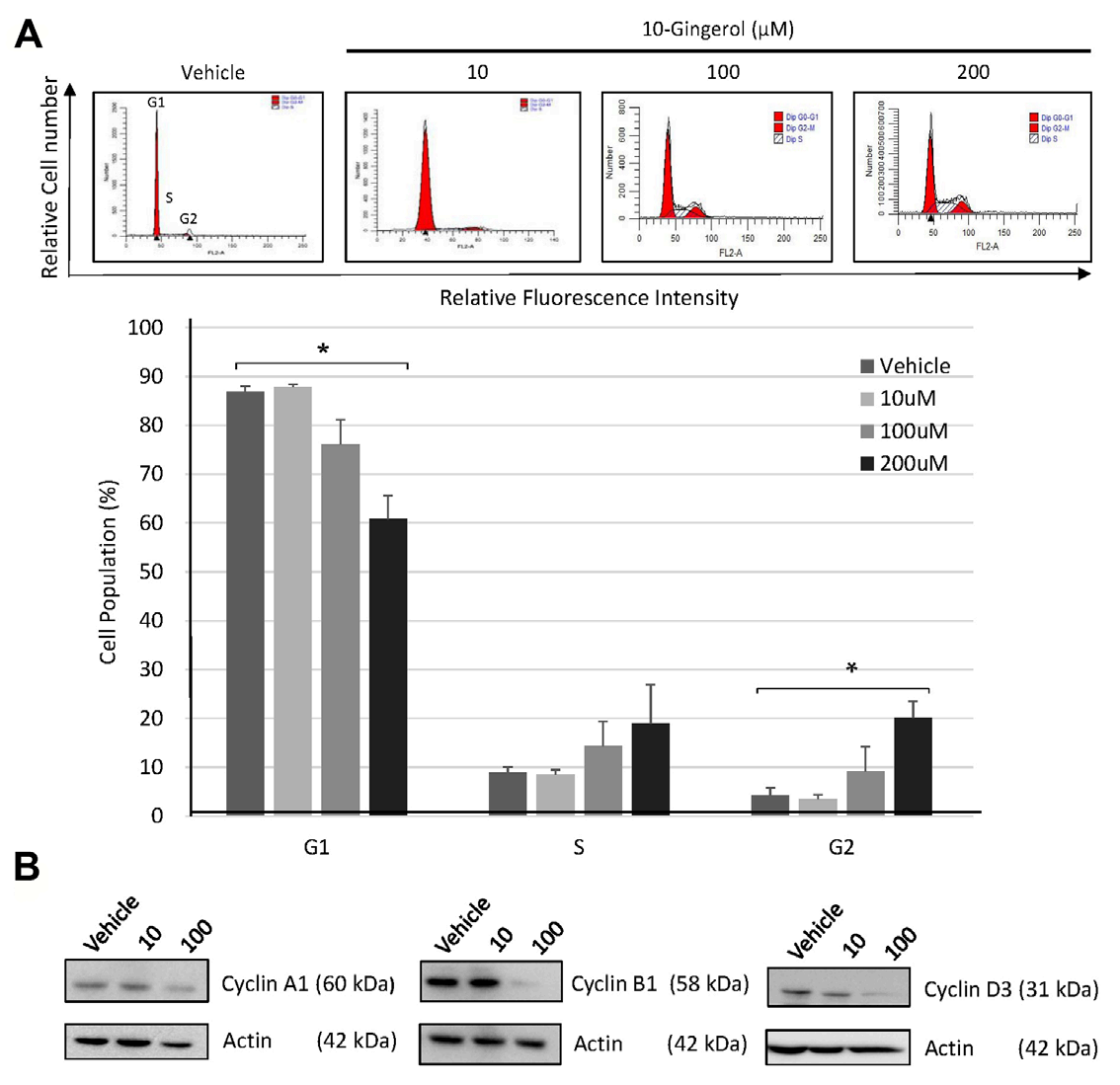
G2 arrest and reduced cyclin A, B1 and D3 expression in 10-gingerol-treated ovarian cancer cells. (**A**) HEY cells were cultured for 72 h in the absence or presence of 10-gingerol, then stained with PI for flow cytometric analysis of DNA content. Flow cytometry histograms are representative of 3 independent experiments. Data shown are the mean ± SEM; asterisk denotes p < 0.05 compared to the vehicle control. (**B**) HEY cells were cultured for 48 h in the absence or presence of the indicated concentrations of 10-gingerol, then cell lysates were prepared and analyzed by western blotting for cyclin A, B1 and D3 expression, as well as β-actin expression to confirm equal loading of protein. Blots shown are representative of 3 independent experiments.

We next performed cell cycle analysis and assessed cell cycle protein levels in HEY cells cultured with or without 10-gingerol. Figure 4A shows that following 72 h exposure to 200 µM 10-gingerol, the percentage of G2 phase cells was increased (20 ± 4 % in 10-gingerol-treated cultures vs. 4 ± 1% in vehicle-treated cultures, *P* < 0.05), while the proportion of cells in G1 was decreased (60 ± 5 % in 10-gingerol-treated cultures vs. 86 ± 1% in vehicle-treated cultures, *P* < 0.05). There are conflicting reports of S phase and G1 phase arrest in breast cancer cells following exposure to 10-gingerol.^7,12^ However, to our knowledge, this is the first report of 10-gingerol-induced G2 growth arrest. Western blot analysis (Figure 4B) revealed decreased expression of cyclin A, B1, and D3 proteins in HEY cells after 48 h treatment with 10-gingerol. Cyclin A is involved in G2 to M phase transition, as well as cell cycle progression through S phase, cyclin B1 regulates mitosis, and D type cyclins are required for G1 entry.^13^ Reduced expression of cyclin A accounts for the accumulation of 10-gingerol-treated HEY cells in the G2 phase. The decreased proportion of 10-gingerol-treated HEY cells in G1 phase is likely due to the combined effects of decreased cyclin B1 and D3 expression.

## Conclusion

We demonstrate here, for the first time, that the natural phenolic compound, 10-gingerol, suppresses ovarian cancer cell growth. The cytostatic effect of 10-gingerol was the result of G2 phase cell cycle arrest. The reduced growth of ovarian cancer cells in the presence of 10-gingerol supports further investigation for the possible use of 10-gingerol in the management of ovarian cancer; however, oral administration of ginger extract (up to 2 g daily), while well tolerated by healthy humans, yields only 1.5 µM 10-gingerol in plasma,^14^ which is well below the amount needed to suppress ovarian cancer cell growth. Any future clinical application will therefore require the development of effective delivery modalities such as intravenous injection of 10-gingerol-loaded nanoparticles, which has already been shown to enhance the therapeutic effect of curcumin.^15^

## Ethical Issues

Not applicable.

## Conflict of Interest

The authors declare that they have no conflict of interests.

## Acknowledgments

This work was supported by discretionary funds from the Canadian Cancer Society (CSS)/Canadian Breast Cancer Foundation (CBCF) and the Queen Elizabeth II Foundation, associated with the CSS/CFCF Endowed Chair in Breast Cancer Research.
